# The 4717C > G polymorphism in periplakin modulates sensitivity to EGFR inhibitors

**DOI:** 10.1038/s41598-019-38742-0

**Published:** 2019-02-20

**Authors:** Hui Mei Lee, Gregory Michael Kelly, Nur Syafinaz Zainal, Pei San Yee, Muhammad Zaki Hidayatullah Fadlullah, Bernard Kok Bang Lee, Chai Phei Gan, Vyomesh Patel, Sok Ching Cheong

**Affiliations:** 1 Head and Neck Cancer Research Team, Cancer Research Malaysia, No. 1, Jalan SS12/1A, 47500 Subang Jaya, Selangor Malaysia; 2 Department of Oral & Maxillofacial Clinical Sciences, Faculty of Dentistry, University of Malaya, Kuala Lumpur, Malaysia

## Abstract

The use of EGFR inhibitors on oral squamous cell carcinoma (OSCC) as monotherapy yielded modest clinical outcomes and therefore would benefit from biomarkers that could predict which patient subsets are likely to respond. Here, we determined the efficacy of erlotinib in OSCC cell lines, and by comparing sensitive and resistant lines to identify potential biomarkers. We focused on the 4717C > G polymorphism in periplakin (PPL) where the CC genotype was associated with erlotinib resistance. To validate this, erlotinib-resistant cell lines harbouring CC genotype were engineered to overexpress the GG genotype and vice versa. Isogenic cell lines were then studied for their response to erlotinib treatment. We demonstrated that overexpression of the GG genotype in erlotinib-resistant lines sensitized them to erlotinib and inhibition of AKT phosphorylation. Similarly, the expression of the CC genotype conferred resistance to erlotinib with a concomitant increase in AKT phosphorylation. We also demonstrated that cell lines with the CC genotype generally are more resistant to other EGFR inhibitors than those with the GG genotype. Overall, we showed that a specific polymorphism in the PPL gene could confer resistance to erlotinib and other EGFR inhibitors and further work to evaluate these as biomarkers of response is warranted.

## Introduction

Oral squamous cell carcinoma is one of the top ten cancers among men in the world^1^. It is most prevalent in India, Bangladesh and Pakistan due to the practice of known risk habits such as smoking, excessive alcohol consumption and betel quid chewing^2^. Patients diagnosed at early stage can be treated by surgery or radiotherapy alone while concurrent radio-chemotherapy is often used in patients with locally advanced disease^3^. About a third of the patients will progress into metastatic stage, with palliative chemotherapy as the only therapeutic option. Recently, pembrolizumab^4^ and nivolumab^5^ have been approved for patients with metastatic OSCC with disease progression during or after chemotherapy. Despite this advancement, therapies for advanced OSCC remains limited and targeted therapies are actively being explored to improve the survival of OSCC patients.

OSCC has been characterized by high expression of epidermal growth factor receptor (EGFR)^6^. Increased activity of EGFR results in activation of downstream signalling cascade such as PI3K/PTEN/AKT, ERK, and Jak/STAT pathways to promote cell proliferation, invasion and metastasis. Hence, increased protein expression of EGFR is a prognostic marker for poor survival in OSCC patients^6^. To target EGFR for therapeutic purposes, inhibitors have been developed and several of these have been tested in OSCC^7^. The success of cetuximab, a recombinant monoclonal antibody targeting EGFR in extending the progression-free survival (PFS) in patients with recurrent/metastatic OSCC, resulted in its approval by US Food and Drug Administration (FDA) in 2006^7^. Whilst this is encouraging, this success has not been recapitulated with small molecule inhibitors targeting EGFR. One of these small molecule inhibitors is erlotinib or known as OSI-774 or Tarceva. Erlotinib is an orally active small molecule that blocks EGFR-mediated intracellular signalling by binding competitively to the ATP binding region^8^. It is approved for the treatment of patients with locally advanced or metastatic non-small cell lung cancer (NSCLC) with stable disease after standard platinum-based first-line chemotherapy^9^. A clinical study showed the efficacy of erlotinib by tumour shrinkage in 9 out of 35 locally advanced OSCC patients in a neoadjuvant setting before surgery^10^. However, a further phase II clinical trial on OSCC patients from 2006 to 2011 did not demonstrate a significant increase in PFS when erlotinib is combined with cisplatin and radiotherapy^11^. Similar results were also shown using another EGFR inhibitor, gefitinib^12^. Despite the conclusions, detail analysis showed that 52% of patients treated with erlotinib and cisplatin had a complete response as compared to 40% of patients who responded to cisplatin alone^11^; for gefitinib, 12.5% of patients who received docetaxel and gefitinib showed response as compared to 6.2% for patients treated with docetaxel alone^12^.

Recent clinical trials on small molecule inhibitors were proven to be more effective when the patients were stratified based on biomarkers. For example, the approval of trametinib and dabrafenib for melanoma patients with BRAF V600 mutations^13^ and olaparib for breast cancer patients who are HER-2 negative and carrying BRCA mutations^14^. Studies in NSCLC showed that 60–80% of the patients with EGFR mutations respond well to erlotinib, but it was evident that patients without these mutations also benefited from erlotinib^15^, suggesting that EGFR is not a reliable biomarker that could predict for drug response. Furthermore, EGFR mutations are not frequently observed in OSCC and hence may not be a useful biomarker in this context^16^. Further biomarker analysis examining the mutational status of EGFR and KRAS, copy number of EGFR and protein expression of EGFR, cMET, HER2, HER3 and PTEN from the TORCH trial in patients with advanced NSCLC was not able to identify other biomarkers for erlotinib^17^ that could be further tested in the OSCC setting. In OSCC, Martins and colleagues attempted to investigate the efficacy of erlotinib on human papillomavirus (HPV) positive tumour by examining p16 expression, but the test was performed in less than 50% of the patients, rendering it difficult for further analysis^11^. Taken together, there is currently no biomarker identified for EGFR inhibitors for OSCC, and this has in part stalled the advancement of these targeted therapies in OSCC^7^.

In order to progress with further testing of EGFR inhibitors, it is pivotal to identify biomarkers that could predict the response to these drugs. In this study, we combined genomic information on cancer cell lines with drug response data to identify potential biomarkers that could predict response to EGFR inhibitors. Focusing primarily on the EGFR inhibitor erlotinib, we demonstrated that a single nucleotide polymorphism (SNP) in PPL could inhibit AKT phosphorylation and modulate response to erlotinib.

## Results

### Effect of erlotinib in OSCC

**Figure 1:**
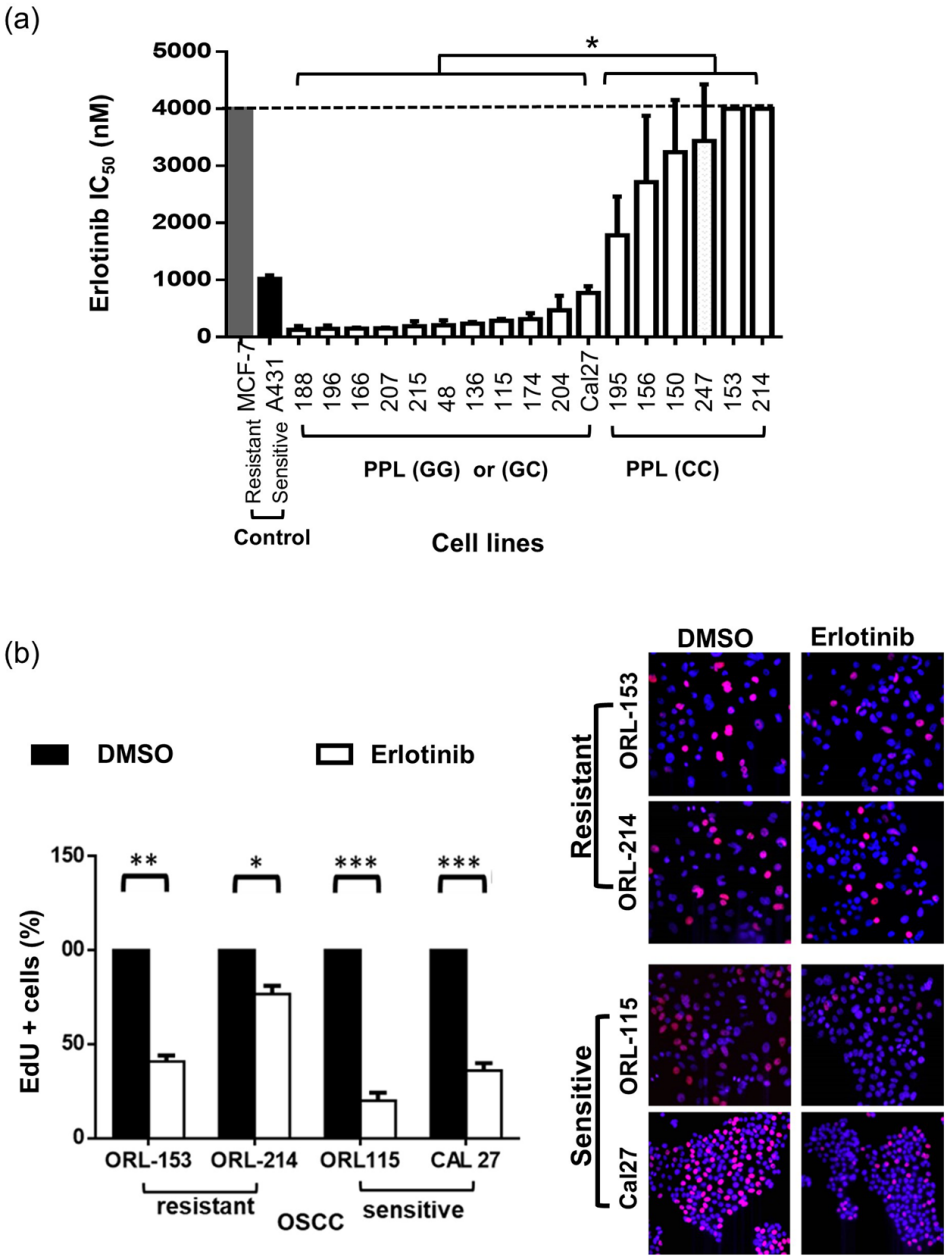
Effect of erlotinib on OSCC. (**a**) IC_50_ of OSCC cell lines towards erlotinib treatment with controls A431 (sensitive) and MCF-7 (resistant) at 72 h. 4 µM was the maximal concentration tested and marked by the dotted line. (**b**) Anti-proliferation effect of erlotinib at 1 µM was demonstrated on sensitive lines, Cal27 and ORL-115, but less evident in resistant lines, ORL-153 and ORL-214. EdU positive cells stained with Alexa Fluor 647 represents actively proliferating cells (pink), DAPI staining (blue) shows all viable cells, 20×. The graphs are the representative results of 2 experimental repeats in comparison to 0.01% DMSO. Error bars represent SEM for the replicates tested in 2 independent experiments. * denotes p < 0.05, ** denotes p < 0.01, *** denotes p < 0.005.

The efficacy of erlotinib in OSCC cell lines was determined by MTT assays. We demonstrated that 11/17 cell lines (ORL-188, -196, -166, -207, -215, -48, -136, -115, -174, -204 and Cal27) were sensitive to erlotinib with IC_50_ at sub-micromolar concentrations (0.15 ± 0.02 µM to 0.62 ± 0.14 µM) and 6 cell lines were resistant with IC_50_ from 1.39 ± 0.05 µM to >4 µM. A431 with IC_50_ less than 1 µM was used as sensitive control^18^ whereas MCF7 was used as a resistant control with IC_50_ of more than 4 µM^19^ (Fig. 1a).

Further, we determined the cytostatic effect of erlotinib on 2 representative cell lines from the erlotinib sensitive and resistant groups respectively by measuring the active DNA synthesis using the EdU assay. We showed that the proliferation rate of sensitive cell lines (Cal27 and ORL-115) reduced significantly by >75% (p < 0.001) when treated with 1 µM erlotinib for 24 hours compared to the control (0.01% DMSO). Meanwhile a large proportion of the cells in the resistant group were still proliferating at 1 µM (44.2% in ORL-153 (*p* = 0.019) and 78.3% in ORL-214 (*p* = 0.011); Fig. 1b).

### Characteristics of erlotinib-sensitive and resistant OSCC lines

We analysed RNA-Seq data from cell lines that are sensitive to erlotinib and the ones that are resistant^20^ to identify unique mutations that could distinguish these groups. We identified a list of candidate genes that might have a role in conferring sensitivity towards erlotinib in OSCC lines, and polymorphisms in the PPL gene (rs1049205, 1766G > A and rs2037912, 4717C > G) were significantly associated with increased sensitivity towards erlotinib treatment (*p* < 0.001 for both). Previous studies have suggested that the linker domain of PPL could bind to AKT^21^. Therefore, we decided to focus on rs2037912 harbouring homozygous GG at codon 4717, coding for glutamine, or homozygous CC, coding for glutamate located in this domain. The polymorphism was confirmed using Sanger sequencing on the cell lines (Supplementary Fig. 1) as well as from the corresponding blood gDNA of the cell line donors (data not shown) indicating that these mutations were not culture-induced. The data showed that of the 17 OSCC cell lines tested, 5 were homozygous GG (ORL-166, -188, -207, -215 and Cal27), 6 were heterozygous GC (ORL-48, -115, -136, -174, -196 and -204) and the remaining 6 were homozygous CC (ORL-150, -153, -156, -195, -214 and -247; Supplemental Fig. 1). Our data showed that cells harbouring the GG and GC genotypes have significantly lower IC_50_ levels compared to cell lines which have the CC genotype (Fig. 1a). Two OSCC cell lines each from the resistant (ORL-214 and ORL-153) and sensitive group (Cal27 and ORL-115) were selected for further experiments.

### Knock-down of PPL re-sensitizes erlotinib-resistant OSCC lines to erlotinib

**Figure 2:**
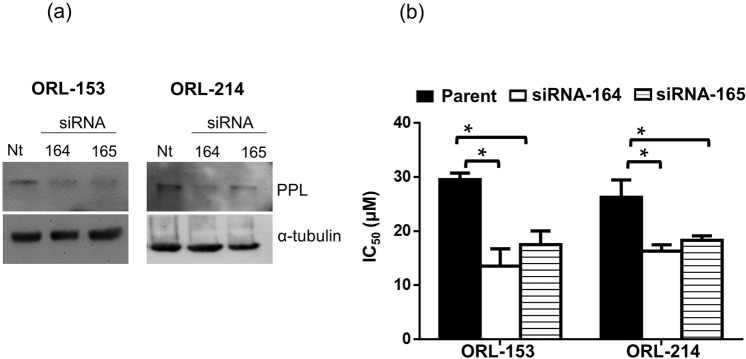
Effect of knocking down PPL expression on IC_50_ of erlotinib-resistant OSCC lines. (**a**) Western blot analyses show the reduction of PPL protein expression by siRNA. (**b**) IC_50_ of erlotinib-resistant cell lines towards erlotinib treatment after upon PPL knock-down. Original blots retaining at least six band widths above and below the band can be found in Supplementary Fig. 5. Bar graphs in (**b**) are means ± SEM from 2 independent experiments conducted in triplicate. Statistical significance p < 0.05 denoted by *.

In order to validate the importance of the PPL gene in modulating the response of OSCCs lines to erlotinib, transient knockdown using siRNA was carried out in erlotinib-resistant lines (ORL-153 and ORL-214). The success of PPL knockdown was validated by western blotting as shown in Fig. 2a where PPL expression was markedly reduced in ORL-153 and ORL-214 cells transfected with siRNA-164 or siRNA-165. Using MTT assays, we demonstrated that reduction of PPL expression using both siRNAs consistently sensitized ORL-153 and ORL-214 to erlotinib with a ~2-fold reduction in the IC_50_ (ORL-153 (*p* = 0.016); ORL-214 (*p* = 0.043); Fig. 2b)_,_ demonstrating that PPL is very likely to be important in modulating response to erlotinib.

### Overexpression of the GG genotype re-sensitizes erlotinib-resistant OSCCs to erlotinib

**Figure 3:**
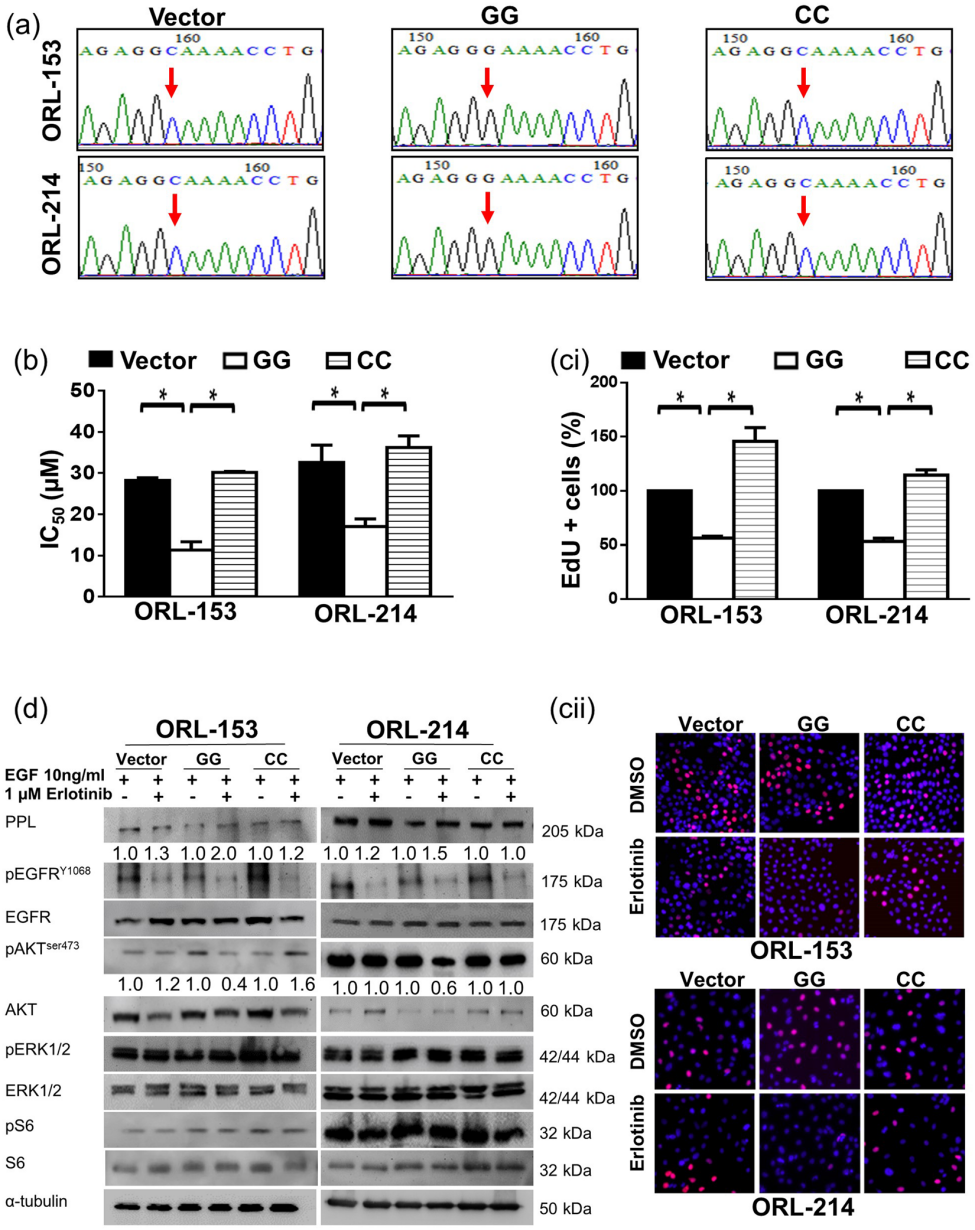
Effect of erlotinib on erlotinib-resistant lines transfected to express PPL[G/G]. OSCC lines resistant to erlotinib harbouring PPL[C/C] were transfected with pCDNA3.1 vector control, pCDNA3.1/PPL[G/G] or pCDNA3.1/PPL[C/C]. (**a**) Electropherogram showed the successful transfection of PPL[G/G] and PPL[C/C] in ORL-153 and ORL-214. (**b**) IC_50_ of ORL-153 and ORL-214 transfected with PPL[G/G] was approximately 2 folds lower than cells transduced with vector control or PPL[C/C]. (**ci**) The proliferation of ORL-153 and ORL-214 transfected with PPL[G/G] were reduced with erlotinib treatment as compared to vector control or PPL[C/C]. Bar graphs in (**b**) and (**ci**) are means ± SEM from 2 independent experiments conducted in triplicate. Statistical significance p < 0.05 denoted by * (**cii**) Representative diagrams of proliferation assays from ORL-153 and ORL-214 cells stained with EdU (pink) and DAPI (blue) as compared to control, 20×. (**d**) Western analysis showed that erlotinib reduced the expression of pAKT^ser473^ in PPL[G/G] as compared to vector control or PPL[C/C]. Densitometry quantification compared protein expression in control and erlotinib-treated group. Original blots retaining at least six band widths above and below the band can be found in Supplementary Figs 6 and 7.

Since cell lines harbouring the GG genotype in PPL are more sensitive to erlotinib, we went on to confirm the effects of this genotype in response to erlotinib. To do this, we overexpressed GG in ORL-153 and ORl-214 which originally carry the CC genotype. The same lines were also transfected with the vector control (VC) as a control and the plasmid expressing the CC genotype to ensure that our observations were not due to the transfection. The success in transfection was confirmed with Sanger sequencing using DNA from the transfected clones (Fig. 3a). With these, we then investigated the change in sensitivity to erlotinib between clones carrying the GG, CC and VC using MTT assays.

**Figure 4:**
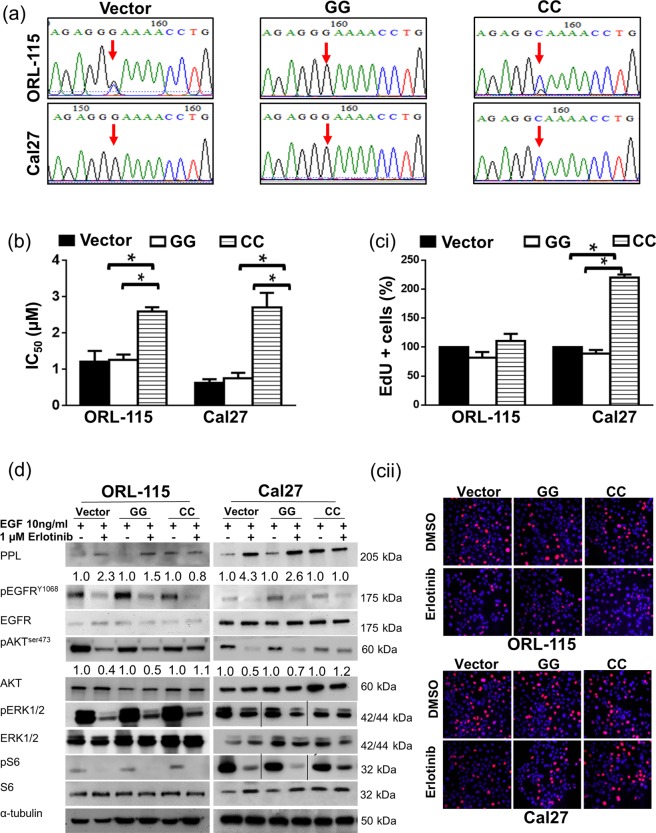
Effect of erlotinib on erlotinib-sensitive OSCC lines transfected to express PPL[C/C]. OSCC lines sensitive to erlotinib harbouring PPL[G/G] were transfected with pCDNA3.1 vector control, pCDNA3.1/PPL[G/G] or pCDNA3.1/PPL[C/C]. (**a**) Electropherogram showed the successful transfection of PPL[G/G] and PPL[C/C] in ORL-115 and Cal27. (**b**) IC_50_ of ORL-115 and Cal27 transfected with PPL[C/C] was approximately 2–4 folds higher than cells transduced with vector control or PPL[G/G]. (**ci**) The proliferation of ORL-115 and Cal27 transfected with PPL[C/C] were less responsive to erlotinib treatment as compared to vector control or PPL[C/C]. Bar graphs in (**b**) and (**ci**) are means ± SEM from 2 independent experiments conducted in triplicate. Statistical significance p < 0.05 denoted by * as compared to control. (**cii**) Representative diagrams of proliferation assays from ORL-115 and Cal27 cells stained with EdU (pink) and DAPI (blue), 20x. (**d**) Western analysis showed that erlotinib does not alter the expression of pAKT^ser473^ in PPL[C/C] as compared to vector control or PPL[G/G]. Densitometry quantification compared protein expression in control and erlotinib-treated group. Original blots retaining at least six band widths above and below the band can be found in Supplementary Figs 8 and 9.

The MTT data showed that the GG genotype sensitized both ORL-153 and ORL-214 to erlotinib treatment (Fig. 3b). Compared to the IC_50_ of ORL-153 (VC) at 29.02 ± 1.7 µM, the IC_50_ of ORL-153 (GG) was reduced by ~3 fold to 11.67 ± 2.1 µM (*p* = 0.015). Similarly, IC_50_ of ORL-214 (GG) was ~2-fold lower than ORL-214 (VC), at 13.1 ± 1.4 µM and 27.6 ± 1.9 µM respectively (Fig. 3b; *p* = 0.048). Overexpressing the CC genotype in ORL-153 and ORL-214 did not alter the IC_50_ significantly in comparison to VC as these lines originally harbour the CC genotype (Fig. 3b). While we had shown that the cytotoxic effect of erlotinib was enhanced by overexpressing the GG genotype in OSCC cells harbouring CC, we were interested to determine if the GG genotype affected the anti-proliferative effect of erlotinib. EdU assay was carried out to analyze the DNA synthesis of the VC, GG, and CC clones of ORL-153 and ORL-214 when treated with 1 µM of erlotinib for 24 hours. Using the proliferation rate of VC treated with 1 µM of erlotinib for 24 hours as control, we demonstrated that the proliferating cells in ORL-153 and ORL-214 overexpressing GG was reduced by ~50%, whilst no significant changes were observed in cells expressing CC in comparison to VC (Fig. 4ci,ii; ORL-153 (*p = *0.025); ORL214 (*p* = 0.040). Biochemical analyses were done to determine the changes of expression in PPL and the molecules within the EGFR signalling pathway in VC, GG, and CC clones after being treated with erlotinib (Fig. 3d). When comparing cells treated with erlotinib with no treatment, the expression level of PPL in GG clone increased by 2-fold in ORL-153 and 1.5-fold in ORL-214 whereas no changes were observed in VC and CC clones. Besides, erlotinib treatment reduced pAkt^ser473^ levels by 2.5-fold and 1.7-fold in GG clones of ORL-153 and ORL-214 respectively in line with the increase response to erlotinib seen in the GG clones, but not in the VC and CC clones. By contrast, the expression of pS6 and pERK1/2 were not affected in VC, GG, and CC clones when treated with erlotinib (Fig. 3d).

### Overexpression of CC confer resistance to erlotinib-sensitive OSCC lines

Having demonstrated that overexpressing the GG genotype confers sensitivity to erlotinib we next investigated the impact of overexpressing the CC genotype in erlotinib-sensitive cells, Cal27 and ORL-115 harbouring the GG genotype. Similarly, Cal27 and ORl-115 were transfected with VC, GG, and CC plasmids. Sanger sequencing confirmed the successful transfections as shown in Fig. 4a. For ORL-115, clones overexpressing CC had IC_50_, 2-fold higher than VC (2.5 ± 0.2 µM and 1.2 ± 0.3 µM respectively; *p* = 0.049). In Cal27, overexpressing the CC genotype increased the IC_50_ of VC by 4.4-fold, from 0.62 ± 0.1 µM to 2.7 ± 0.4 µM (Fig. 4b; *p* = 0.037). Consistently, the CC genotype conferred resistance to erlotinib where EdU assays demonstrated that in ORL-115, EdU expressing cells were higher in CC than VC clones, albeit not statistically significant. For Cal27, the cells expressing CC were less responsive to erlotinib, as evidenced by higher proliferating cells as compared to VC (Fig. 4ci,ii; *p* = 0.028). Western blot analyses were carried out to determine the biochemical changes in EGFR signalling in VC, GG, and CC after being treated with erlotinib. Protein expression of PPL was increased by 2.3-fold and 1.5-fold respectively in VC and GG clones of ORL-115 when treated with erlotinib. For Cal27, PPL expression increased by 4.3-fold and 2.6-fold respectively in VC and GG clones upon erlotinib treatment. Erlotinib treatment did not alter the level of PPL in CC clones. Phosphorylation of AKT levels were not changed in the CC clones of ORL-115 and Cal27 despite erlotinib treatment as compared to VC and GG clones where pAKT^ser473^ were reduced as clearly demonstrated in the western blot analysis (2-fold and 2.5-fold in VC and GG of ORL-115 cells respectively; 2-fold and 1.4-fold in VC and GG of Cal27 cells respectively). Both pERK1/2 and pS6 expression levels were reduced in VC, GG and CC clones upon erlotinib treatment (Fig. 4d).

### Effect of polymorphism in PPL on other EGFR inhibitors

To investigate if a polymorphism in PPL could affect the response of OSCC to another EGFR TKIs, we carried out MTT to determine the IC_50_ of OSCC when treated with afatinib, a pan-EGFR inhibitor. As shown in Supplementary Fig. 2, the mean IC_50_ of cell lines harbouring GG genotype was significantly lower than cell lines with CC genotype upon afatinib treatment (*p* = 0.017). To validate the hypothesis that genotype GG and CC would modulate the response to EGFR TKIs in other head and neck cancer (HNSC) cell lines, we looked into the Genomics of Drug Sensitivity in Cancer (GDSC)^22^. We were able to analyse data on HNSC cell lines treated with afatinib and gefitinib from the database. As for afatinib, the mean IC_50_ of cell lines with GG genotype was 12.36 µM ± 18.76, lower compared to cells harbouring CC genotype with IC_50_ at 17.37 µM ± 27.6 for afatinib treatment even though it was not statistically significant (Supplementary Fig. 3). Similarly, the mean IC_50_ of cell lines with GG genotype had IC_50_ of 0.22 µM ± 0.15, lower compared to cell lines harbouring CC genotype with IC_50_ at 2.08 µM ± 3.5 for gefitinib treatment (Supplementary Fig. 4). Due to the limited HNSC cell lines treated with erlotinib in GDSC, we were not able to make a comparison using this data.

## Discussion

Gene-drug interaction databases such as Cancer Therapeutics Response Portal (CTRP) and GDSC facilitates the identification of putative biomarkers as drug responses can be correlated to the well-characterized genomic features^23^. Leveraging on a panel of well-characterized OSCC cell lines we set out to identify putative biomarkers for erlotinib. Here, we identified polymorphisms in PPL that is predictive of erlotinib response in OSCC. PPL is a cornified envelope precursor and a member of the plakin family, with high homology to the other members of the plakin family. PPL functions to provide strong support to terminally differentiated keratinocytes, acts as an epidermal barrier^24^, and helps to re-organized keratin intermediate filament network to stimulate wound closure^25^. PPL was found to be significantly associated with resistance to sunitinib^26^, a multi-target receptor tyrosine kinase, in cell lines derived from various types of cancer. However, further work to validate how PPL may confer resistance to sunitinib has not been pursued. Here, for the first time, we demonstrate that a specific polymorphism that occurs in the linker domain of PPL that binds to AKT modulates response to erlotinib and other tyrosine kinase inhibitors^21^.

An increase in sensitivity to erlotinib was observed when PPL was knocked-down by siRNA, however, the IC_50_ remained relatively high. Although these data suggest that PPL could modulate sensitivity to erlotinib, the modest difference could be due to the fact that the level of PPL knock-down was not sufficient to sensitize cells to erlotinib to the IC_50_ levels seen in inherently sensitive cells. Perhaps, the level of PPL may not be the only factor driving erlotinib sensitivity, and other factors such as its ability to interact with other proteins depending on its genotype could also be an important factor. Notwithstanding, our data is supported by Tonoike and his colleagues^27^ who showed that knocking down PPL reduced cell proliferation in pharyngeal cancer. This demonstration that the disruption of endogenous PPL levels could affect response to erlotinib suggests that PPL is involved in EGFR signalling. To examine this in more detail, we identified and validated the role of a polymorphism at rs2037912 on PPL in modulating sensitivity to erlotinib. We focused on rs2037912 where cell lines with the CC genotype (a glutamine at amino acid 1573) were significantly associated with resistant to erlotinib compared to cell lines with a glutamate (GG) at the same position. This polymorphism is particularly interesting in the context of EGFR signalling because previous immunoprecipitation studies have indicated that codon 4717 resides in a section of the PPL protein that binds to AKT^21^. To demonstrate whether the PPL polymorphism would directly affect erlotinib sensitivity, we engineered the GG genotype in erlotinib-resistant cells endogenously carrying CC genotype and showed that the change could sensitize cells that were originally resistant to erlotinib. This response was accompanied by a reduction in the expression of pAKT^ser473^ rendering the GG clones more sensitive to erlotinib treatment. Meanwhile, overexpression of the CC genotype into erlotinib responsive cells endogenously harbouring GG genotype sustained the expression of pAKT^ser473^ and cells were less responsive to erlotinib. Our data clearly demonstrated that this particular polymorphism in PPL directly affects response to erlotinib and this is likely due to modulating the activation of AKT, a key signalling molecule downstream of the EGFR. We noted that although overexpressing GG into erlotinib-resistant cells significantly reduced the IC_50_ when treated with erlotinib, however the IC_50_ was still relatively high and this could be possibly due to the effects of the second polymorphism (rs1049205), which changes the amino acid position at 589 from arginine (GG) to glutamine (AA) that was also significantly associated with erlotinib sensitivity. We did not test the effect of both polymorphisms simultaneously in this study so that the effect of each polymorphism can be delineated independently for their effects on drug sensitivity. Both of the loci have high LOD score and codon change from GG to AA at codon position 1766 occur together with CC change to GG at codon position 4717 in cell lines sensitive to erlotinib (Supplementary Table 1), indicating that further investigation into these polymorphisms and their role in conferring drug resistance is warranted.

In addition to erlotinib, we also found the CC genotype at rs2037912 to be associated with resistance to other EGFR inhibitors including afatinib. Afatinib is approved for metastatic NSCLC with common EGFR mutations as well as L861Q, G719X, and/or S768I^28^. Regardless of the working mechanism of the inhibitor, the activation of AKT plays an important role in the downstream signalling in the EGFR pathway. In acquired resistance to EGFR inhibitors, pAKT^ser473^ level was elevated in lung cancer, and increased pAKT^ser473^ level was suggested as a predictive biomarker for EGFR tyrosine-kinase inhibitors (TKIs) response^29^. PPL which interacts with AKT, acts as a localization signal to AKT or a shuttle for delivery of AKT to the various cellular compartments^21^. For example, when PPL localizes to the cell membrane, AKT becomes activated and translocated to other compartments in the cell^30^, further underscoring that fact that PPL could play an important role in affecting the treatment response by EGFR TKIs.

Clinical trials on NSCLC patients have demonstrated a better response to EGFR inhibitors amongst patients of Asian ethnicity compared to Caucasian patients^31,32^. Whilst one of the factors is that Asian populations have a higher mutational rate in EGFR^33^, this could also be due to other genetic factors. Looking at the PPL polymorphism in the HapMap study of Asian and Caucasian populations (available from dbSNP website)^34^, ~47% of Asians including Japanese and Han Chinese in Beijing have the GG genotype as compared to only ~27% of Europeans. By contrast, ~23% of Caucasian have CC genotype in comparison to ~8.5% in Asians. The effect of this polymorphism in PPL between Asian and Caucasian populations could provide some additional insights into the differential response to EGFR inhibitors and should be investigated further.

One of the limitations of the study is the lack of suitable *in vivo* models to further investigate the role of 4717C > G polymorphism in PPL in modulating sensitivity to erlotinib. Cal27 overexpressing CC or GG in PPL were transplanted into NOD.CB17-Prkdcscid/NCrCrl mice but at the end of the study, genotyping showed that overexpression was not retained. Future investigations on establishing a suitable model to overexpress GG or CC stably and how the polymorphism alters the structure of PPL and affect binding to AKT is warranted. Nevertheless, our data add to the array of possible biomarkers for EGFR targeted therapy and suggest that other proteins that interact with molecules within the EGFR signalling pathway could confer resistance to treatment. In conclusion, our data provide evidence that a polymorphism in PPL could play a role in conferring resistance to EGFR inhibitors by affecting the activation of AKT. This finding is significant because many other tyrosine kinase receptors signal through AKT, and PPL could also play a role in conferring resistance in these pathways. We show that proteins such as PPL that interacts with signalling molecules could confer resistance to kinase inhibitors and affords an opportunity to investigate PPL as a biomarker for cancer therapeutics that directly or indirectly inhibits AKT phosphorylation.

## Materials and Methods

### Reagents and materials

Erlotinib HCl (Selleckchem, TX, USA) was dissolved in dimethyl sulfoxide (DMSO; Sigma) at a stock concentration of 10 mM and diluted in culture medium for use. Afatinib (LC Laboratories, Woburn, MA, USA) was dissolved in DMSO at a stock concentration of 40 mM, and further diluted in culture medium to the required concentrations. Primary antibodies used in these studies were EGFR (#2232), pEGFR^Y1068^ (#2234), AKT (#2938), pAKT^ser473^ (#4060), ERK1/2 (#9102), pErk1/2 (#9101), S6 ribosomal protein (#2317), pS6 ribosomal protein (#2211; Cell Signaling Technology), periplakin (clone EPR8296; ab131269; Abcam) and α-tubulin (T5158; Sigma). The secondary antibodies goat anti-rabbit IgG-HRP (SB4010-05) and goat anti-mouse IgG-HRP (SB1010-05) were purchased from Southern Biotech. 3-(4,5-dimethylthiazol-2-yl)-2,5-diphenyltetrazolium bromide (MTT; Sigma-Aldrich) solution was prepared at 5 mg/ml in phosphate-buffered saline (PBS).

### Cell culture

The ORL cell lines used were established in our laboratory as described previously^20^. These cell lines were cultured in Dulbecco’s Modified Eagle’s Medium/Nutrient mixture F-12 HAM’s medium (DMEM/F-12; Hyclone) supplemented with 500 ng/ml hydrocortisone (Sigma-Aldrich), and 10% heat-inactivated fetal bovine serum (FBS; Gibco). The breast cancer cell line MCF7 was cultured in RPMI 1640 medium (Gibco) supplemented with 10% FBS and 100 IU Penicillin/Streptomycin. Cal27 and A431 were cultured in Dulbecco’s Modified Eagle’s Medium High Glucose (DMEM-HG, Gibco) with 10% heated-inactivated FBS and 100 IU Penicillin/Streptomycin. Cell line authentication was carried out by comparing the STR profiles of cell lines to the matched blood or tumour from the patients for 16 ORL lines^20^ or to ATCC database for other lines. Cell lines were routinely checked for mycoplasma using MycoAlert™ Mycoplasma Detection Kit (Lonza). All cultures were incubated in a humidified atmosphere with 5% CO_2_ at 37 °C.

### MTT Cell Viability Assay

Cell viability of OSCC lines was determined using MTT assay as described previously. Briefly, 2–5 × 10^4^ cells were seeded per well in 96-well plates and treated with erlotinib at ~1 nM to 4 µM. 72 hours after treatment, MTT solution was added to each well and incubated at 37 °C for 3 hours. Next, 100 µL of dimethyl sulfoxide (DMSO) was added to dissolve the purple formazan and the optical density was determined using Biotek microplate reader set at 570 nm. IC_50_ of the cell lines was presented as the mean ± SEM of three independent experiments with DMSO (0.01%) as the control. MCF7 which is resistant to erlotinib^19^ and A431 previously reported to be sensitive^18^ to erlotinib were used as resistant and sensitive controls.

### Click-iT™ EdU Proliferation Assay and Cell Imaging

Two OSCC cell lines shown to be resistant to erlotinib in this study (ORL-214 and ORL-153) and two other sensitive lines (Cal27 and ORL-115) were subjected to cell proliferation assay using Click-iT EdU Alexa Fluor 647 Imaging Kit (Invitrogen) according to manufacturer’s protocol. Briefly, cells were plated on coverslips in 12-well plates and treated with erlotinib at 1 µM for 24 hours. Following EdU incubation at 10 µM for 2 to 4 hours at 37 °C as optimized previously for each cell line, cells were fixed with 3.7% (v/v) formaldehyde in PBS for 15 minutes and permeabilized using 0.5% (v/v) Triton-X in PBS for 20 minutes at room temperature. Cells were washed with 3% (w/v) BSA in PBS. Next, the cells were stained with the Click-iT reaction cocktail (1X Click-iT reaction buffer, CuSO_4_, Alexa Fluor azide, reaction buffer additive) for 30 minutes in the dark. After washing (3% BSA), cells were incubated with 1X Hoechst 33342 solution (5 μg/mL) for 30 minutes. Coverslips were mounted with VECTASHIELD mounting medium (Vector Laboratories Inc) after washing with PBS. Olympus IX81 imaging system equipped with XcellencePro imaging software (Olympus) was used to capture EdU positive cells with Alexa Fluor 647: excitation 650 nm and emission 667 nm while live cells stained with Hoechst were captured using a filter with excitation at 360–370 nm and emission at 420 nm at 20x magnification. Images were merged using XcellencePro imaging software. Unmerged images were analyzed using Quick Count^®^ to determine the number of Edu positive cells and live cells (Tiong *et al*., 2016; manuscript submitted). Percentage of EdU positive cells was calculated using the following formula: (EdU positive cells/number of DAPI cells) × 100. The percentage of EdU-positive cells was normalized against vehicle control. The proliferation rate of cell lines was presented as the mean ± SEM of two independent experiments with DMSO (0.01%) as the control.

### Bioinformatics Pipeline Analysis of the 17 OSCC cell lines

Identified mutations from the OSCC cell lines were tested for statistical significance between the erlotinib sensitive (IC_50_ less than 1 µM as described in^35^ and resistant groups using Fisher’s exact test. Briefly, for each single nucleotide variants (SNV) identified, the SNV was transformed into a two-way contingency table with two categories (i) sensitive and (ii) resistant. Cell lines ORL (188, 196, 166, 207, 215, 48, 136, 115, 174, 204, Cal27) were grouped as sensitive and ORL (195,156,150,247,153,214) were grouped as resistant. Fisher’s exact test was performed in R (version 3.20) using the function “fisher.test”. SNV with *p* value < 0.05 was considered significant.

### Transient siRNA transfection

The expression of PPL in OSCC cells was inhibited by transfecting cells with 50 nM MISSION siRNA against PPL (SASI_Hs01_00025164, SASI_Hs01_00025165) or non-targeting control (Sigma-Aldrich) using HiPerfect transfection reagent (Qiagen) according to the manufacturer’s instructions. Cells were collected 48 hours post-transfection to be plated for MTT assay and western blotting.

### Stable expression of the PPL[GG] and PPL[CC] in OSCC cell lines

The pcDNA3.1 FLAG plasmids construct carrying the PPL CC or GG genotype respectively at position 4717 were purchased from GenScript. Plasmids were transfected into the ORL-153, ORL-214, ORL-115 and Cal27 using Turbofect transfection reagent (Fermentas) according to the manufacturer’s instructions. Control cells were transfected with the pCDNA3.1 FLAG vector. Transfected cells were selected using G418 (Sigma-Aldrich) at 400 µg/ml for 7 days.

### Sequencing

Genomic DNA (gDNA) was extracted using the Blood and Cell Culture DNA Kit (Qiagen) from the following samples: blood genomic DNA from donors of the ORL cell lines, ORL cell lines, Cal27, ORL-214, ORL-153, ORL-204, ORL-115 transfected with PPL[G/G], PPL[C/C]. Polymerase chain reaction (PCR) was conducted to amplify exon 22 of the PPL gene using the primers: PPL-seq-F 5′ACCGAGCAAGAGATCCAGAG3′ and PPL-seq-R 5′TGTGTCAGGGTGGATGACTACG3′. PCR was set up in 50 µl containing 1X amplification buffer (containing 1.5 mM MgCl_2_), 100 µM of each dNTP, 50 µM of each primer and 1.25 U of Taq DNA polymerase (Promega) using 200 ng of gDNA template. PCR was performed using Veriti DNA thermocycler (Applied Biosystems) with an initial denaturation at 95 °C for 3 minutes, followed by 30 cycles of denaturation at 95 °C for 30 seconds, primer annealing at 55 °C for 30 seconds and DNA extension at 72 °C for 30 seconds. The PCR product was subjected to 1% agarose gel electrophoresis and viewed using FluorChem^TM^ HD2 imaging systems (Alpha Innotech). The 462 bp fragment was purified using QIAquick Gel Extraction Kit (Qiagen) and sequenced using Sanger sequencing.

### Western Blotting

Cells were lysed on ice using RIPA lysis buffer (20 mM Tris, pH 8.0, 150 mM NaCl, 10% glycerol, 1% Nonidet P-40 (NP40), 1 mM HALT proteinase and phosphatase inhibitors (Thermo Scientific). Proteins were collected from untreated cells to establish basal levels of protein expression as well as treated cells. Treated cells were serum starved for 16 hours before treatment with 1 µM of erlotinib (or DMSO control) in serum-free medium for 1 hour before the cells were washed and stimulated with 10 ng/ml of recombinant human EGF (R&D Systems Inc) for 10 minutes before harvesting. Protein concentration was measured using BCA protein assay (Thermo Scientific). 20 µg of protein was resolved in 8% SDS-polyacrylamide gels and transferred to a polyvinylidene fluoride membrane (PVDF; Millipore) in a tank transfer apparatus for 2 hours at 100 V (Bio-Rad, Hercules, USA). The membrane was blocked with 5% (w/v) skim milk in Tris-buffered saline with 1% (w/v) tween 20 (TBST) for 1 hour and probed with primary antibodies at 1:1000 in 1% BSA in TBST overnight at 4°C. Blots were washed in TBST (x3 for 5 minutes). The blots were then incubated with secondary antibodies conjugated with horseradish peroxidase (dilution 1:10000). After washing using TBST, detection was performed by ECL™ detection reagents (GE Healthcare, Piscataway, NJ) and viewed using FluorChem^TM^ HD2 imaging systems (Alpha Innotech). Blots were exposed for 1 minute to detect the bands except for PPL, pEGFR^Y1068^, pS6 and S6, where blots were exposed for 3 minutes. To normalize the loading, tubulin was used as loading control. Densitometry analyses of protein bands were performed using Image J^36^ and normalized by α-tubulin. Fold-change of band intensity was normalized by comparing to DMSO-treated control.

### Statistical Analysis

Statistical analyses were performed using Graph Pad Prism (GraphPad Software). Data are presented as mean ± SEM of at least 2 independent experiments conducted in triplicate. Unpaired t-test with two-tailed was used to calculate statistical significance between control and treatment groups. ANOVA Kruskal-Wallis test was carried out to determine the significance between control and more than one treatment groups. Significant *p* value was indicated as *p* < 0.05, *p* < 0.01 and *p* < 0.005.

## Supplementary information

Supplementary information

## Data Availability

The datasets generated during and/or analysed during the current study are available from the corresponding author on request.
