# Supplementary material for: The 4717C > G polymorphism in periplakin modulates sensitivity to EGFR inhibitors
Source: Sci Rep. 2019 Feb 20;9:2357. doi: 10.1038/s41598-019-38742-0 (PMC6382785; doi:10.1038/s41598-019-38742-0)
Supplement: Supplementary file 1 — Supplementary information [file 41598_2019_38742_MOESM1_ESM.pdf]

The 4717C>G polymorphism in periplakin modulates sensitivity to EGFR inhibitors

Hui Mei Lee [1], Gregory Michael Kelly [1], Nur Syafinaz Zainal [1], Pei San Yee [1], Muhammad Zaki Hidayatullah Fadlullah [1], Bernard Kok Bang Lee [1], Chai Phei Gan [1], Vyomesh Patel [1] and Sok Ching Cheong [1, 2]*

[1]Head and Neck Cancer Research Team, Cancer Research Malaysia, No. 1, Jalan SS12/1A, 47500 Subang Jaya, Selangor, Malaysia

[2]Department of Oral & Maxillofacial Clinical Sciences, Faculty of Dentistry, University of Malaya, Kuala Lumpur, Malaysia

*Correspondence: Sok Ching Cheong

Address: Cancer Research Malaysia, No. 1, Jalan SS12/1A, 47500 Subang Jaya, Selangor, Malaysia; Telephone: +603 2712 3224 ; Fax: +603 2712 3225

Email: sokching.cheong@cancerresearch.my

Supplementary Table 1: Codon of PPL at 4717 (rs2037912) and position 1766 (rs1049205) in OSCC cell lines.

						Sensitive						Resistant					
Codon	188	196	166	207	215	48	136	115	174	204	Cal27	195	150	247	153	214	156
4717	GG	GC	GG	GG	GG	GC	GC	GC	GC	GC	GG	CC	CC	CC	CC	CC	CC
1766	AA	GA	AA	AA	AA	GA	GA	GA	GA	GA	AA	GG	GG	GG	GG	GG	GG

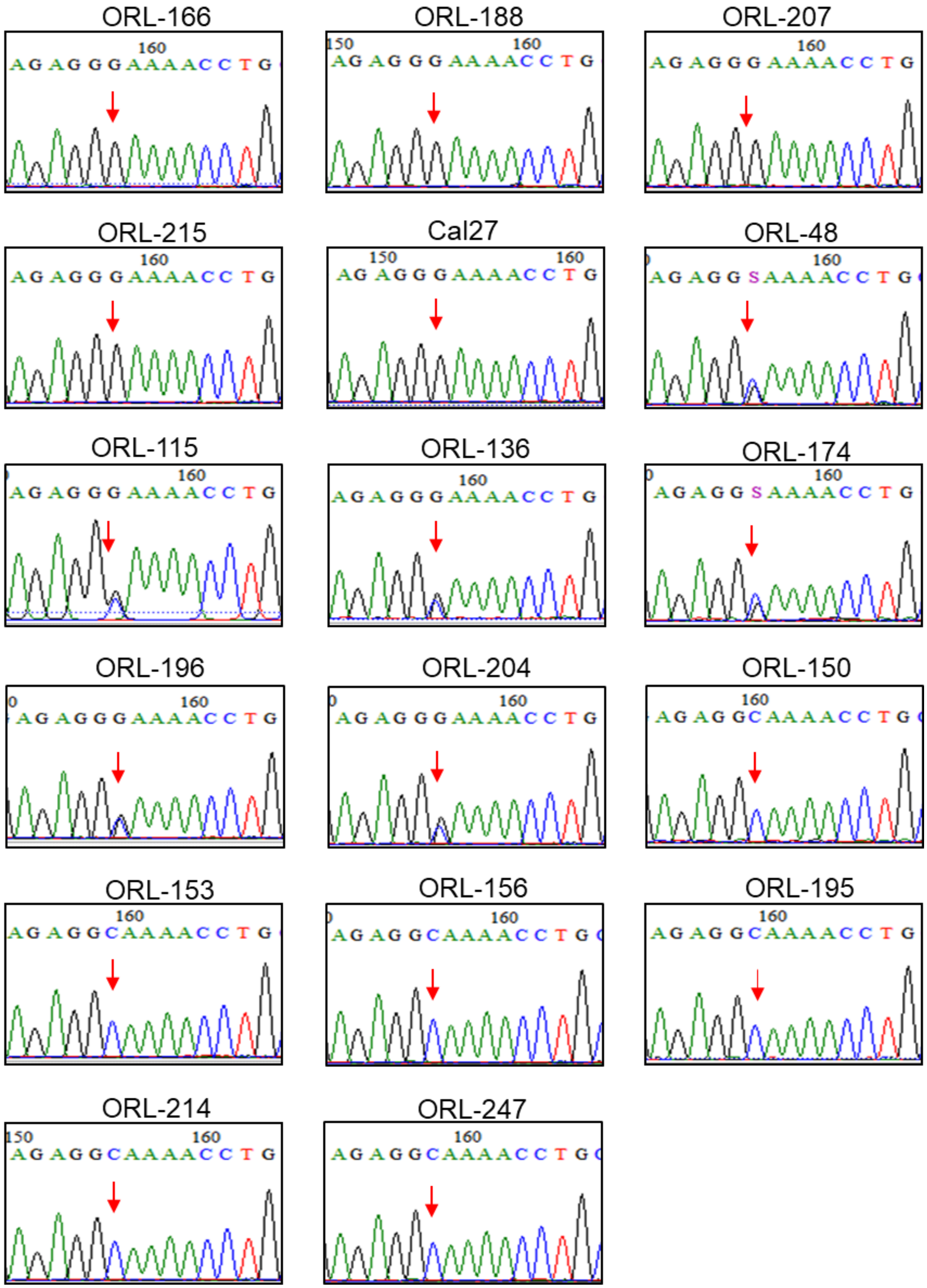

Supplementary Figure 1. Polymorphism status of PPL in OSCC lines. Electropherograms show the OSCC cell lines harbouring either [G/G], [C/C] or [G/C] at 4717 in the PPL gene.

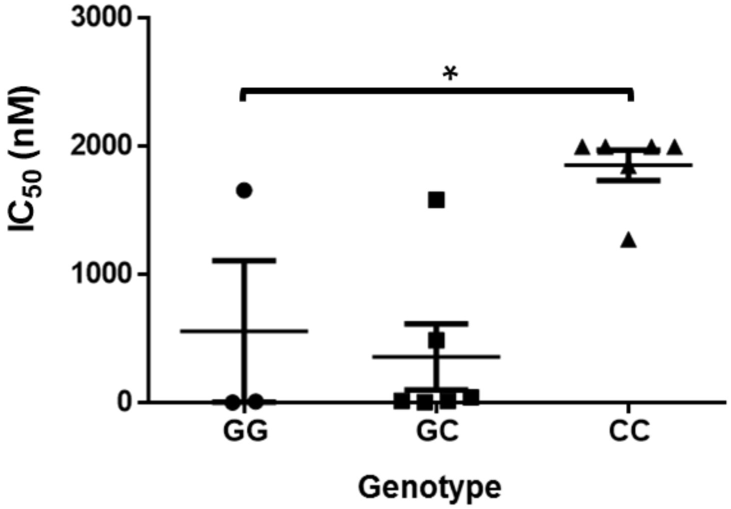

Supplementary Figure 2. IC_{50} of OSCC cell lines towards afatinib treatment at 72 h.

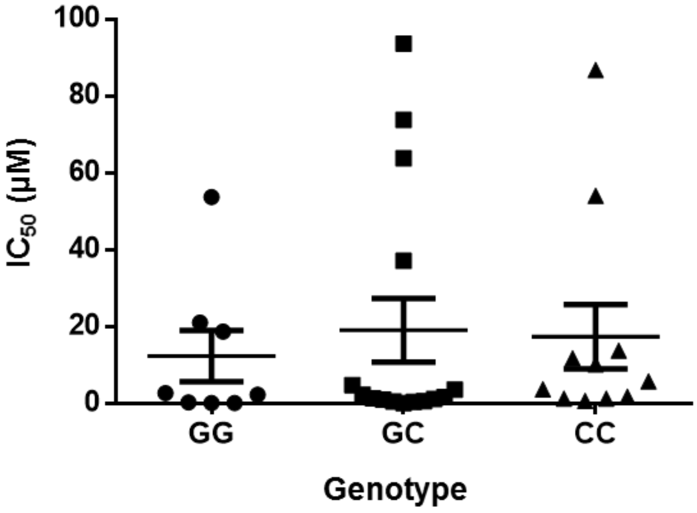

Supplementary Figure 3. IC_{50} of HNSC cell lines towards afatinib treatment as reported in GDSC.

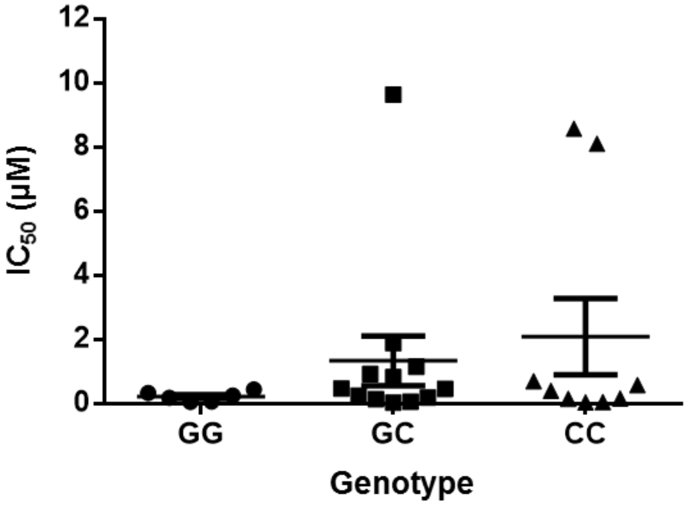

Supplementary Figure 4. IC_{50} of HNSC cell lines towards gefitinib treatment as reported in GDSC.

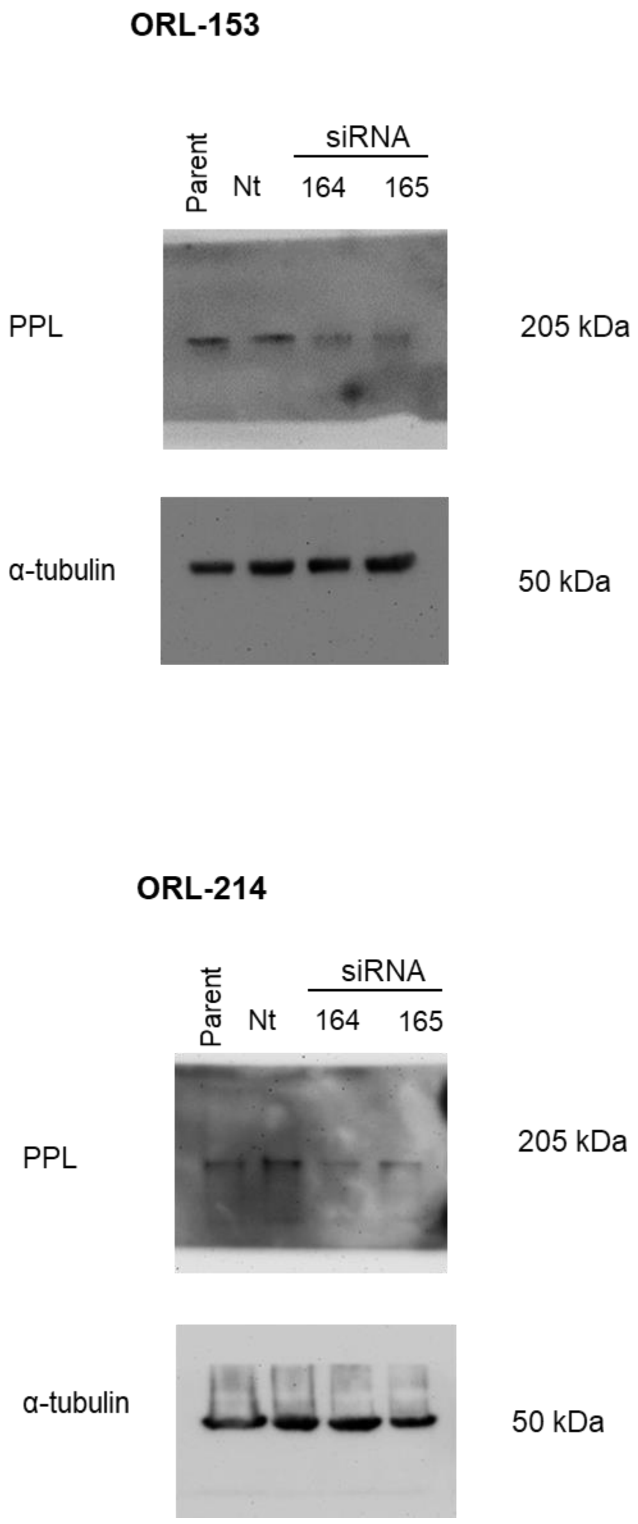

Supplementary Figure 5: Original western blots of PPL-knock down in ORL-153 and ORL-214 retaining at least six band widths above and below the band.

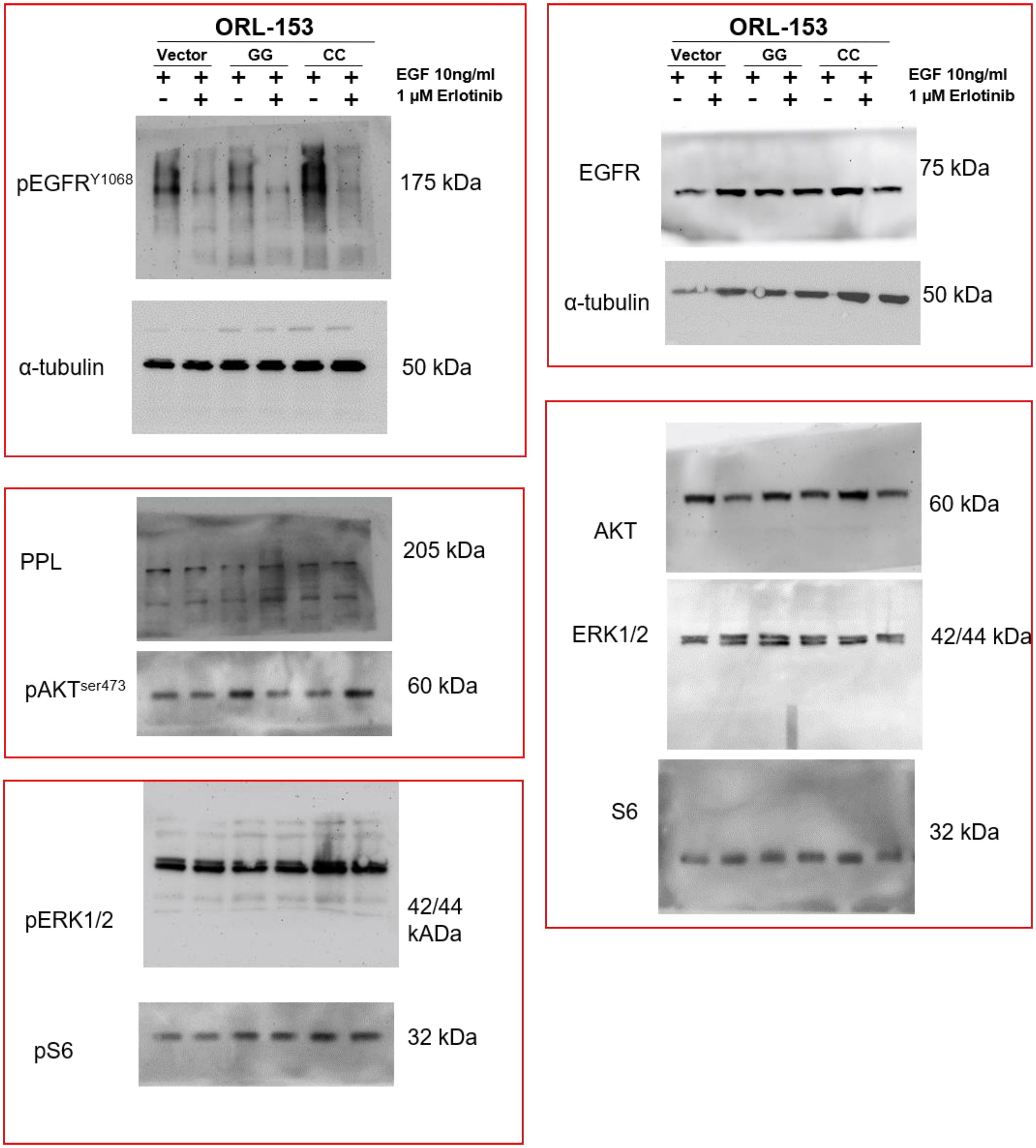

Supplementary Figure 6: Original western blots of ORL-153 retaining at least six band widths above and below the band.

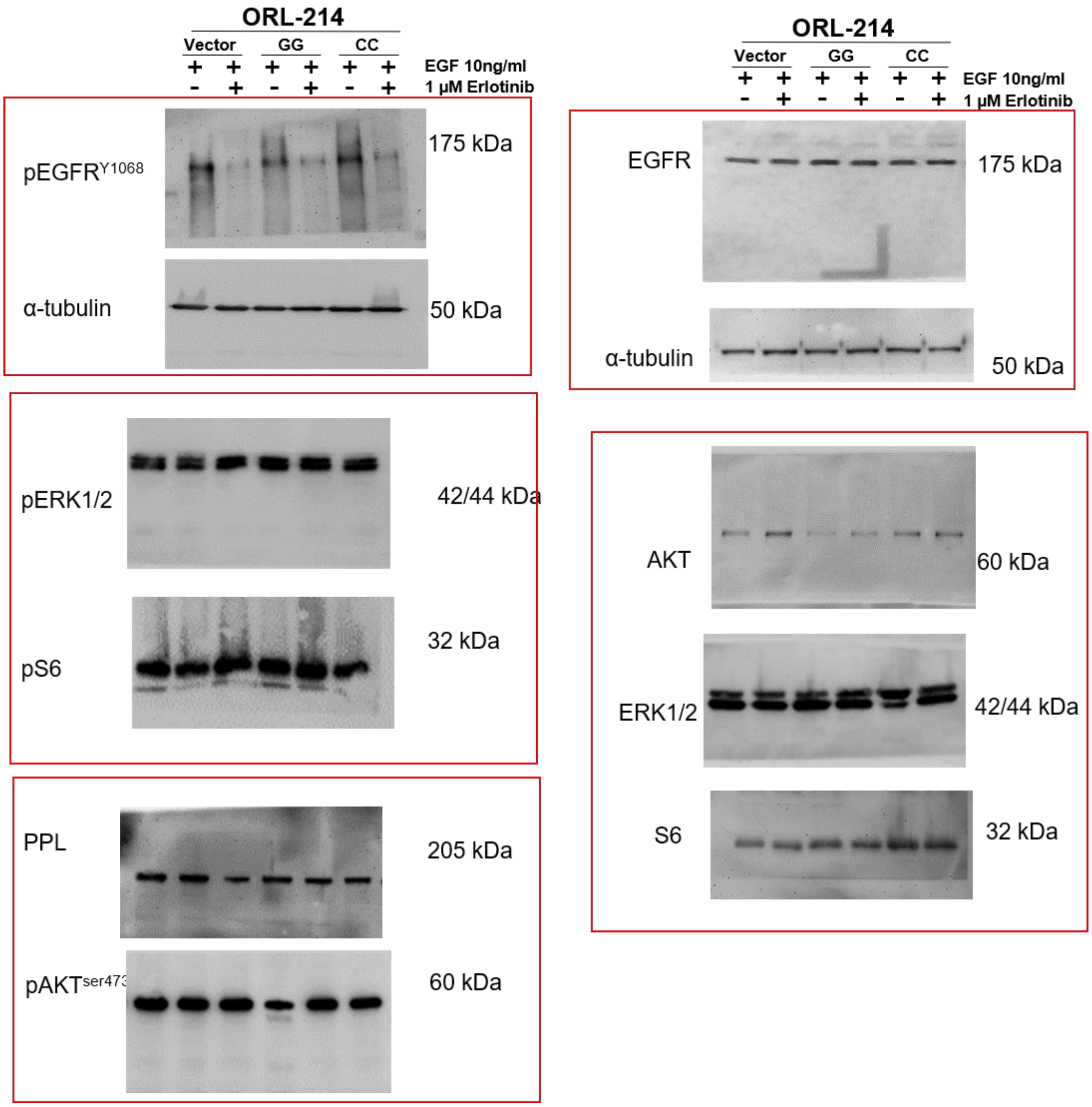

Supplementary Figure 7: Original western blots of ORL-214 retaining at least six band widths above and below the band.

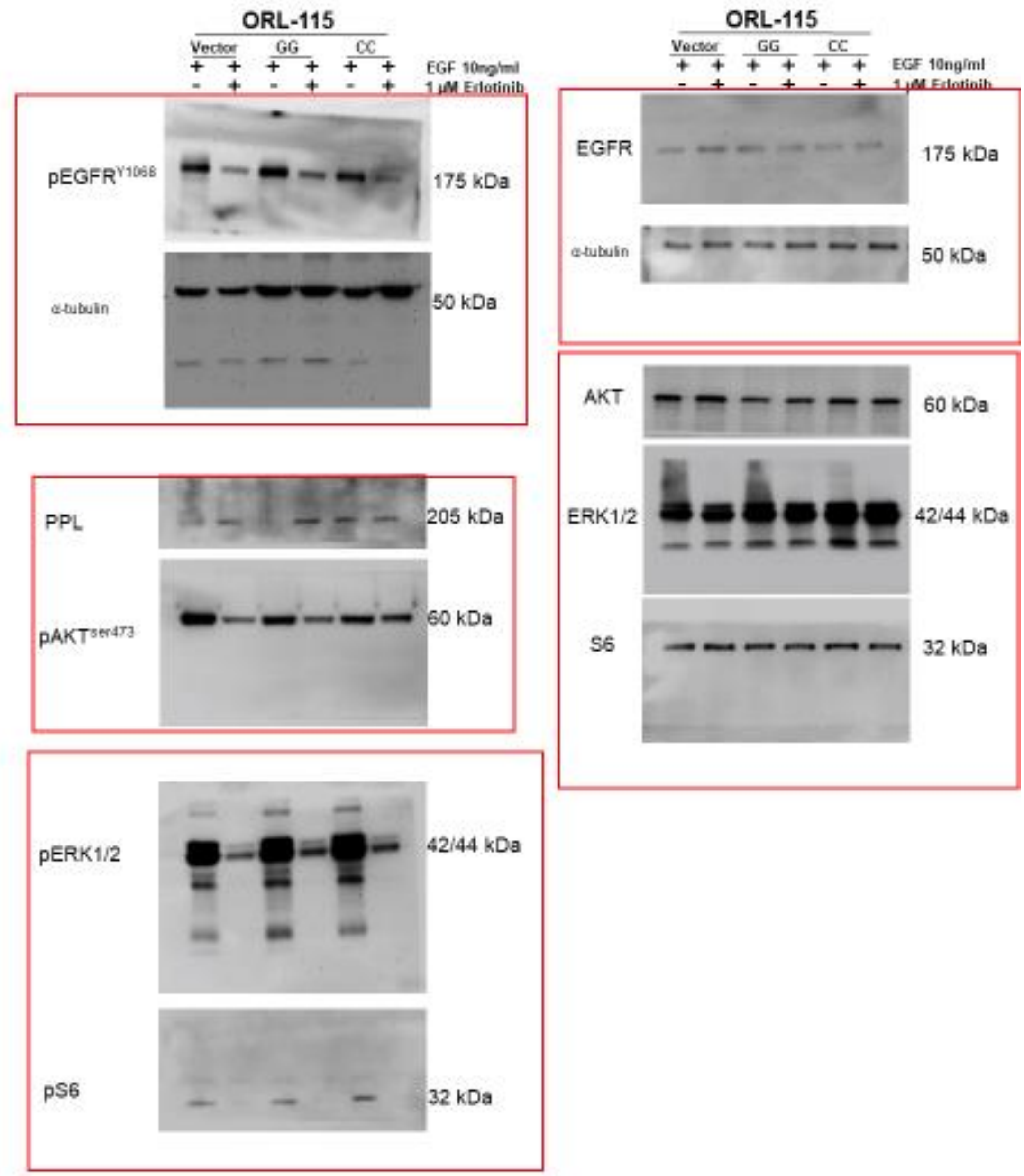

Supplementary Figure 8: Original western blots of ORL-115 retaining at least six band widths above and below the band.

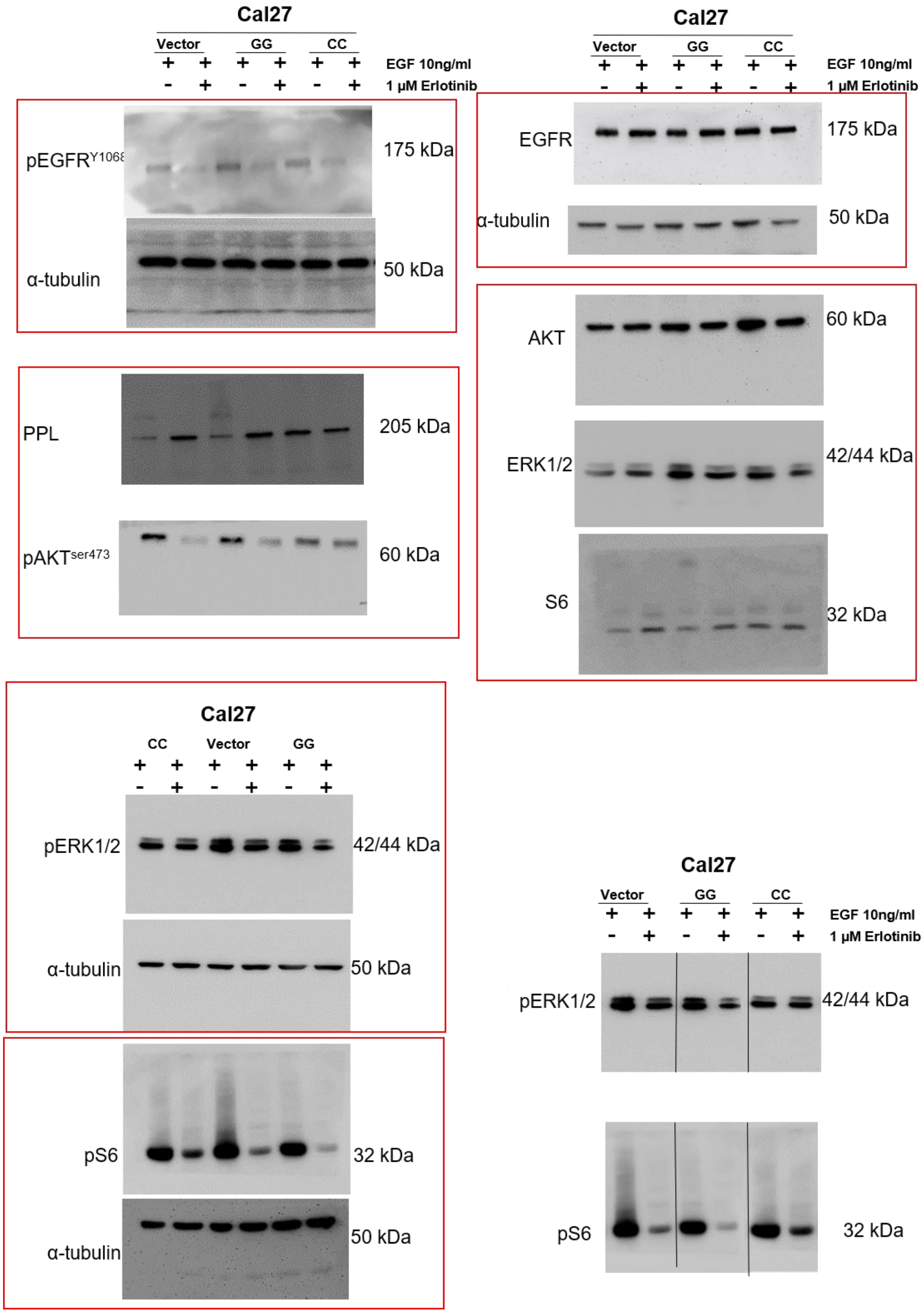

Supplementary Figure 9: Original western blots of Cal27 retaining at least six band widths above and below the band.
